# A hope for ineffective antibiotics to return to treatment: investigating the anti-biofilm potential of melittin alone and in combination with penicillin and oxacillin against multidrug resistant-MRSA and -VRSA

**DOI:** 10.3389/fmicb.2023.1269392

**Published:** 2024-02-01

**Authors:** Saba Jalalifar, Shabnam Razavi, Rasoul Mirzaei, Gholamreza Irajian, Kamran Pooshang Bagheri

**Affiliations:** ^1^Microbial Biotechnology Research Center, Iran University of Medical Sciences, Tehran, Iran; ^2^Department of Microbiology, School of Medicine, Iran University of Medical Sciences, Tehran, Iran; ^3^Venom and Biotherapeutics Molecules Lab., Department of Medical Biotechnology, Biotechnology Research Center, Pasteur Institute of Iran, Tehran, Iran

**Keywords:** MRSA, VRSA, biofilm, AMPs, melittin, penicillin, oxacillin, synergism

## Abstract

**Background:**

The emergence and rapid spread of multi-drug resistant (MDR) bacterial strains, such as methicillin-resistant *Staphylococcus aureus* (MRSA) and vancomycin-resistant *S. aureus* (VRSA), have posed a significant challenge to the medical community due to their ability to form biofilm and develop resistance to common antibiotics. Traditional antibiotics that were once effective in treating bacterial infections are now becoming increasingly ineffective, leading to severe consequences for patient outcomes. This concerning situation has called for urgent research to explore alternative treatment strategies. Recent studies have shown that antimicrobial peptides (AMPs) hold promise as effective agents against biofilm-associated drug-resistant infections as well as to enhance the efficacy of conventional antibiotics. Accordingly, we aimed to investigate the antimicrobial and antibiofilm effects of melittin AMP, both alone and in combination with penicillin and oxacillin, against biofilm-forming MDR-MRSA and -VRSA.

**Methods:**

In this study, we investigated the kinetics of biofilm formation and assessed various parameters related to the antimicrobial and antibiofilm efficacy of melittin and antibiotics, both alone and in combination, against MDR-MRSA and -VRSA. The antimicrobial parameters included the Minimum Inhibitory Concentration (MIC), Minimum Bactericidal Concentration (MBC), Fractional Inhibitory Concentration Index (FICi), Fractional Bactericidal Concentration Index (FBCi), and the antibiofilm activity of melittin and antibiotics indicated by the Minimum Biofilm Inhibitory Concentration (MBIC), Minimal Biofilm Eradication Concentration (MBEC), Fractional Biofilm Inhibitory Concentration Index (FBICi), and Fractional Biofilm Eradication Concentration Index (FBECi).

**Results:**

The MIC results showed that all *S. aureus* isolates were resistant to penicillin (≥0.25 μg/mL), and 66% of isolates were resistant to oxacillin. The geometric means of the MIC values for penicillin, oxacillin, and melittin were 19.02, 16, and 1.62 μg/ml, respectively, and the geometric means of the MBC values for penicillin, oxacillin, and melittin were 107.63, 49.35, and 5.45 μg/ml, respectively. The study revealed that the combination indexes of melittin-penicillin and melittin-oxacillin, as determined by FIC values against all isolates, were 0.37 and 0.03, respectively. Additionally, melittin-penicillin and melittin-oxacillin exhibited combination indexes based on FBC values against all isolates at 1.145 and 0.711, respectively. Besides, melittin inhibited the biofilm formation of all *S. aureus* isolates, with MBIC values ranging from 10 to 1.25 μg/mL, and MBEC values ranging from 40 to 10 μg/mL. Generally, the combination indexes of melittin-penicillin and melittin-oxacillin, determined using FBIC values against all isolates, were 0.23 and 0.177, respectively. Moreover, melittin-penicillin and melittin-oxacillin typically had combination indexes based on FBEC values against all isolates at 5 and 2.97, respectively.

**Conclusion:**

In conclusion, our study provides evidence that melittin is effective against both planktonik and biofilm forms of MRSA and VRSA and exhibits significant synergistic effects when combined with antibiotics. These results suggest that melittin and antibiotics could be a potential candidate for further investigation for *in vivo* infections caused by MDR *S. aureus.* Furthermore, melittin has the potential to restore the efficacy of penicillin and oxacillin antibiotics in the treatment of MDR infections. Applying AMPs, like melittin, to revive beta-lactam antibiotics against MRSA and VRSA is an innovative approach against antibiotic-resistant bacteria. Further research is needed to optimize dosage and understand melittin mechanism and interactions with beta-lactam antibiotics for successful clinical applications.

## Introduction

The rise and rapid dissemination of multi-drug resistant (MDR) bacterial strains, including methicillin-resistant *Staphylococcus aureus* (MRSA) and vancomycin-resistant *S. aureus* (VRSA), have become a grave concern for the medical community. These pathogens possess the ability to form biofilms, rendering them even more resilient against conventional antibiotics (Craft et al., 2019). Biofilms are complex communities of microorganisms that attach to surfaces and protect themselves from the immune system and antimicrobial agents (Yin et al., 2019). The emergence of MDR MRSA and VRSA strains has made the treatment of these infections even more challenging (Tarai et al., 2013). When the prevalence of a certain resistance pattern in bacterial infections exceeds a certain threshold, a wider variety of antibiotics and combination medicines may be required for the empirical treatment of MDR infections, which can have adverse effects (van Duin and Paterson, 2016). The formation of biofilms and entrapment of bacterial cells in the polymer-based matrix reduces the sensitivity of bacteria to antimicrobial compounds and the body’s resistance to microorganisms, making it difficult to eradicate infections (Asma et al., 2022). Biofilms are responsible for up to 80% of all bacterial diseases, and finding a feasible solution to the resistance associated with biofilms is crucial (Pokharel et al., 2022). As a result, once-effective antibiotic treatments are losing their efficacy, leading to severe consequences for patient outcomes. This alarming situation necessitates urgent research to explore innovative and alternative treatment strategies to combat antibiotic-resistant infections (Singh et al., 2014). In this regard, monotherapy frequently results in antibiotic resistance in biofilm, and antibiotics should typically used in combination therapy with other antimicrobial agents (Mirzaei et al., 2023). To address this issue, discovering new classes of antibiotics with diverse mechanisms of action has become a top priority (Miethke et al., 2021). Antimicrobial peptides (AMPs), which are the most diverse antimicrobial compounds that have received much attention due to the emergence of resistance in pathogenic bacteria to common antibiotics, have shown promise (Luong et al., 2022). In particular, melittin, a natural AMP found in bee venom, has demonstrated potent antimicrobial activity against MRSA and other bacteria, as well as anti-biofilm properties (Choi et al., 2015).

On the other hand, traditional antibiotics, such as oxacillin and penicillin, have become less effective against MRSA due to the development of resistance mechanisms (Lade and Kim, 2021). To address this challenge, researchers have explored alternative strategies, including the use of AMPs to reactivate antibiotics such as beta-lactams against MRSA.

Melittin has shown a variety of antimicrobial effects *in vitro* and *in vivo* (Choi et al., 2015). In what form? can be used to treat of some local infection such as skin, burn, device associated infections, as well as prevention of post-surgical adhesion. Mirzaei et al. (2022b) found that hemolytic activity of melittin at the concentration of 5 μg/mL was 91.6%, for this reason in this study, we used synergism to reduce the dose of melittin to minimize its erythrolytic effect.

Melittin acts by disrupting the integrity of bacterial cell membranes, leading to bacterial death (Guha et al., 2021). The idea behind combining AMPs with beta-lactam antibiotics is to enhance the effectiveness of the antibiotics against MRSA. Beta-lactam antibiotics work by inhibiting bacterial cell wall synthesis, but MRSA has developed resistance mechanisms, such as the production of beta-lactamase enzymes that break down these antibiotics (Bush and Bradford, 2016). By utilizing AMPs like melittin, which can directly target and kill bacteria, it is possible to overcome this resistance mechanism and re-sensitize MRSA to beta-lactam antibiotics (Al-Ani et al., 2015). It has been found that the antibacterial peptide melittin has a very strong effect on the killing of various pathogens such as *Acinetobacter baumannii*, *Staphylococcus aureus*, *Staphylococcus epidermidis*, and *Pseudomonas aeruginosa* (Jamasbi et al., 2016; Akbari et al., 2019; Mirzaei et al., 2022a,b).

Hence, in the initial phase of this study, the antimicrobial efficacy of melittin was examined alone against the isolated VRSA, MRSA, and MSSA strains. Following this, the study progressed to investigate the potential synergistic interactions between melittin and the penicillin and oxacillin antibiotics. Additionally, based on the aforementioned findings, we conducted a series of experiments to evaluate the antibiofilm capabilities of this peptide against biofilms formed by VRSA, MRSA and methicillin-sensitive *S. aureus* (MSSA)isolates. Finally, we aimed to assess the synergistic effects of combining melittin with penicillin and oxacillin in the context of biofilm.

## Materials and methods

### Media, reagents, and drugs

For the experiments conducted in this study, various antibiotics, media, and reagents were utilized. The antibiotic disks were obtained from MAST (Mast Co., UK), while the powders of oxacillin and penicillin were acquired from Sigma-Aldrich (St. Louis, MO, USA). Media and agar supplies were obtained from Merck (Merck Co., USA). Specifically, mannitol salt agar, blood agar, Mueller-Hinton agar, Mueller-Hinton broth (MHB), sodium chloride (NaCl), and magnesium chloride (MgCl2) were sourced from Merck (Merck Co., USA). Chemical reagents including crystal violet, glucose, and agarose were purchased from Sigma-Aldrich (St. Louis, MO, USA). In addition, 96-well sterile polystyrene, non-treated microplates, available in both flat-bottom and round-bottom variants, were obtained from NEST (Biotechnology Co., Ltd., China), and Jet Biofil (Guangzhou, China).

### Peptide

Melittin (GIGAVLKVLTTGLPALISWIKRKRQQ) was synthesized by DGpeptides Company (Wuhan, Hubei, China) using solid-phase synthesis techniques and ensured purity greater than 96% (Mirzaei et al., 2022b). The purity of the synthetic peptides was verified through reversed-phase high-performance liquid chromatography, and the accuracy of the synthesis was confirmed through liquid chromatography-mass spectrometry (LC-MS) equipment. The peptide content was validated using the bicinchoninic acid assay (BCA) (Bevalian et al., 2021).

### Collection and confirmation of clinical isolates and ATCC strains

All experiments in this study utilized eight strains of *S. aureus*, which included clinical VRSA, MRSA, and MSSA, as well as ATCC strains. Six clinical isolates were obtained from human sources at Motahhari burns hospital in Tehran, Iran (Table 1). Moreover, *S. aureus* ATCC 25923, and *S. aureus* ATCC 29213 were procured from the Pasteur Culture Collection of Tehran, Iran. The isolates were collected from a diverse range of individuals in terms of age and gender, ensuring that there were no duplications; only a single sample per patient was taken. To characterize *S. aureus* isolates, several biochemical tests were employed, including assessments of colony morphology, gram-positive properties, clustering of cocci, catalase activity, mannitol fermentation, DNase production, and coagulase activity (Bevalian et al., 2021). Then PCR amplification was done to confirm the species of the isolates previously identified phenotypically as *S. aureus*. To extract bacterial DNA from staphylococcal strains, a phenol-chloroform technique was employed in the presence of sodium dodecyl sulfate (SDS), preceded by pretreatment with higher doses of lysozyme (200 mg/ml final concentration) and proteinase K (300 mg/ml final concentration). The extracted DNA was subsequently purified using phenol-chloroform-isoamyl alcohol (25:24:1, vol/vol/vol), chloroform-isoamyl alcohol (24:1, vol/vol), and then precipitated with sodium acetate and ethanol at −20°C. The precipitated DNA was washed with 70% ethanol and resuspended in 100 μl of Milli-Q water. PCR amplification of *16srRNA* and *nuc* genes was done (Abbasi-Montazeri et al., 2013; Zhang et al., 2021; Supplementary Table 1). The confirmed isolates were then stored at −70°C in brain heart infusion (BHI) broth with 30% glycerol.

**TABLE 1:** The characterization of *S. aureus* isolates.

Strains	MSSA/MRSA and VSSA	MIC (μg/ml)		Disk			
		Van	Ox	GM	CD	Rif	DXT
1	MSSA/VSSA	2	2	S	R	S	I
2	MRSA/VSSA	2	>256	R	R	R	I
3	MSSA/VSSA	0.5	2	S	S	S	S
4	MRSA/VRSA	128	128	R	R	R	R
5	MRSA/VRSA	32	32	R	R	R	R
6	MRSA/VSSA	2	256	R	R	R	R
ATCC25923	MSSA/VSSA	1	0.5	S	S	S	S

MRSA, methicillin-resistant *S. aureus*; MSSA, Methicillin sensitive *S. aureus*; VRSA, vancomycin- resistant *S. aureus*; Van, vancomycin; Ox, oxacillin; GM, gentamicin; CD, clindamycin; Rif, rifampin; DXT, doxycycline.

### Effects of melittin alone and in combination with antibiotics toward planktonic form

The primary objective of this research was to delve into the antimicrobial attributes of melittin, concentrating particularly on its efficacy against *S. aureus* isolates. Moreover, we investigated the synergistic potential of melittin when combined with penicillin and oxacillin. This investigation aimed to shed light on the remarkable properties of melittin and its cooperative effects with conventional antibiotics in combating *S. aureus* infections.

### Minimum inhibitory concentration, minimum bactericidal concentration and MBC/MIC ratio

To determine the Minimum Inhibitory Concentration (MIC) values of melittin, penicillin, and oxacillin in *S. aureus* isolates, a standard microtiter dilution method was employed using Muller Hinton Broth (MHB) medium, in accordance with Clinical and Laboratory Standards Institute 2021 (CLSI) (Kowalska-Krochmal and Dudek-Wicher, 2021). Fresh colonies of *S. aureus* were grown overnight at 37°C in 5 mL of MHB medium. The quantity of bacterial cells was calibrated to the 0.5 McFarland standard using spectrophotometry at 625 nm, and to improve the accuracy of cell quantification, the optical density (OD)was set at 0.09 in this study, instead of the typical range of 0.08 to 0.1, which is equivalent to 10^8^ CFU/mL. The bacterial cell quantities were then adjusted to 10^6^ CFU/mL in the same medium. In 96-well microplates, 100 μL of MHB medium was added to each well. Melittin (from 40 to 0.039 μg/mL) and antibiotics (from 512 to 1 μg/mL), produced in a normal saline solution, were serially diluted in the microplates. Next, 100 μL of the provided suspension, containing 2 × 10^6^ bacteria, was added to each well of the microplates and incubated at 37°C for 18–24 h, the lowest value of the investigated drugs that completely inhibited observable bacterial growth, was used to determine the MIC, and also, 100 μL of the provided suspension, containing 2 × 10^6^ bacteria, were cultured on MHB medium and counted, with the MBC values identified as the lowest concentrations of agents required to kill 100% of the cultured isolates. The concentration of the peptide or antibiotic that resulted in complete eradication of bacteria was considered as MBC (Thabaut and Meyran, 1984). All experiments were conducted in accordance with CLSI guidelines. Finally, the MBC/MIC ratio was computed as a means of determining whether isolates exhibited tolerance to antimicrobial agents.

### Fractional inhibitory concentration index

In summary, the synergistic effects of melittin, penicillin, and oxacillin were evaluated using the broth microdilution method. The antibacterial agents were serially diluted and added to microtiter plates, followed by the addition of a bacterial suspension. The microplates were incubated for 24 h at 37°C. The fractional inhibitory concentration index (FICi) was calculated for the combined antibacterial agents, using the formula FIC = (MIC drug A in combination/MIC drug A alone) + (MIC drug B in combination/MIC drug B alone). The FIC indices were used to determine the type of drug interaction, with values of *n* ≤ 0.5 indicating synergy, values of 0.5 < *n* < 1 indicating partial synergy, a value of *n* = 1 indicating an additive effect, values of 1 < *n* < 4 indicating an indifferent effect, and values of 4 ≤ n indicating an antagonistic effect (Mirzaei et al., 2023). All experiments were conducted using the broth microdilution method in accordance with established protocols.

### Fractional bactericidal concentration index

To investigate the interactions between antibacterial drugs, the MBC values were used in conjunction with the broth microdilution checkerboard method, also known as the fractional bactericidal concentration index (FBCi). The antibacterial agent interaction method was carried out in a manner similar to the FIC method. A 100 μL volume of the diluted bacterial stock containing 10^5^ CFUs/mL was added to each well, and the microplate was incubated at 37°C for 24 h. After incubation, 10 μL from each well was cultured on MHA, and the MBC values of melittin, penicillin, and oxacillin were determined as the lowest concentrations of agents required to kill 100% of the cultured isolates. Finally, the FBCi was calculated using the formula FBCi = (MBC drug A in combination/MBC drug A alone) + (MBC drug B in combination/MBC drug B alone). The FBCi indices were used to determine the type of interaction between the antibacterial agents, as mentioned for FICi (Mirzaei et al., 2023).

### Biofilm assay and kinetics of biofilm formation

In this study, the microplate assay technique was used to study biofilm formation. Fresh colonies of *S. aureus* ATCC 25923 were cultured overnight at 37°C in 5 mL of TSB with 1% Glucose (Khandan Del et al., 2019). The bacterial suspension’s absorbance was measured at a wavelength of 625 nm to create a 0.5 McFarland standard suspension, with a value of 0.09. A prepared bacterial suspension containing 10^7^ CFUs was created by adding 100 μL of the suspension to 900 μL of TSB under varied conditions in a sterile tube. Next, 200 μL of the bacterial suspension, equivalent to 2 × 10^6^ cells, was added to each well of a 96-well microplate and incubated at 37°C and 70 rpm for 24 h. After incubation, the wells were washed thrice with normal saline and allowed to air dry. Methanol (200 μL) was added to fix the biofilm, and the microplates were allowed to dry at room temperature. Crystal violet (0.05%) was added to each well and incubated for 5 min. The microplates were washed thrice with normal saline and allowed to dry at room temperature. Finally, 200 μL of absolute ethanol was added to each well and shaken at 37°C for 30 min. The contents of each well were transferred to another microplate, and an absorbance measurement was taken at 595 nm using a Synergy HTX Multi-Mode Microplate Reader (BioTek Co., Winooski, VT, USA). All experiments were repeated three times, and the biofilm formation assay based on optimized conditions. The OD cut-off value (ODc) was established using the following formula: ODc = average OD of negative control + (3 × SD of negative control), where the ODc is three standard deviations (SD) above the mean of the OD of the negative control. Finally, the results were sorted into four groups based on their ODs: (1) strong biofilm producer (4 × ODc < OD); (2) medium biofilm producer (2 × ODc < OD ≤ 4 × ODc); (3) weak biofilm producer (ODc < OD ≤ 2 × ODc); and (4) non-biofilm producer (OD ≤ ODc) (Mirzaei et al., 2023).

Besides, to acquire a more precise understanding of biofilm formation in VRSA, MRSA, and MSSA, we conducted a comprehensive analysis of the kinetics involved. It has been established that biofilm formation in *S. aureus* strains is a strain-dependent process, meaning that the development of mature biofilms varies across different strains and over varying periods of time. For a thorough examination, we evaluated the biofilm development kinetics of all isolates for a duration of 48 h (Khandan Del et al., 2019), Considering this information, we proceeded with the biofilm experiments, focusing on evaluating the OD of each sample at specific time intervals: 3, 6, 9, 12, 18, 24, and 48 h. By measuring the OD, we aimed to gain insights into the progression of biofilm formation for each strain. To ensure the reliability and accuracy of our results, we repeated the experiments three times. By employing this rigorous approach, we aimed that would shed light on the intricate processes involved in biofilm formation across various strains of *S. aureus*.

### Antibiofilm effects of melittin alone and in synergy with penicillin and oxacillin

Several studies have provided evidence supporting the disruptive and inhibitory properties of melittin in relation to biofilm formation. Melittin has exhibited promising outcomes in reducing the viability and biomass of biofilms when administered alone. In light of these findings, we conducted experiments to assess the antibiofilm potential of this peptide against biofilms formed by *S. aureus* isolates. Additionally, we evaluated the synergistic effects of melittin in combination with penicillin and oxacillin against *S. aureus* biofilms.

### Minimum biofilm inhibitory concentration

In summary, the inhibitory effects of melittin, penicillin, and oxacillin on pre-formed biofilms were examined by determining the Minimum Biofilm Inhibitory Concentration (MBIC) (Anbarasi et al., 2020). *S. aureus* colonies were grown in TSB medium supplemented with 1% Glu, and the bacterial quantity was adjusted to the 0.5 McFarland standard. A suspension of 2 × 10^6^ CFUs was added to each well of 96-well microplates and incubated for 24 h at 37°C with shaking at 70 rpm. After incubation, the contents of the wells were washed with normal saline solution. Next, 100 μL of melittin (ranging from 40 to 0.039 μg/μL), penicillin, and oxacillin (ranging from 1,024 to 2 μg/μL) were added to the wells at a volume of 100 μL and incubated at 37°C for 24 h, and the quantity of biofilm was measured. The MBIC was determined as the least quantity of melittin and antibiotics that resulted in at least 90% inhibition in biofilm biomass compared to the untreated control. The percentage of biofilm inhibition was calculated using a standard formula. All experiments were repeated three times for each isolate.

### Minimum biofilm eradication concentration

The Minimum Biofilm Eradication Concentration (MBEC) assay was conducted using 96-well microplates, following the previously described protocol with modifications to determine the effectiveness of melittin, penicillin, and oxacillin in killing and degrading bacteria within biofilms (Mirzaei et al., 2022b). As noted previously (Bardbari et al., 2018), the isolates were given 24 h to produce a pre-formed biofilm. The wells were then gently discarded and washed three times with normal saline solution. Next, 100 μL of melittin (ranging from 40 to 0.039 μg/μL), penicillin, and oxacillin (ranging from 1,024 to 2 μg/μL) were added to the wells at a volume of 100 μL and incubated at 37°C for 24 h. The Minimum Biofilm Eradication Concentration (MBEC) assay was performed as previously described by using 96 Well microplates (Mirzaei et al., 2022b). Modifications were made to determine how well melittin, penicillin, and oxacillin could kill bacteria that were surrounded in biofilms as well as degrade them. In summary, as previously noted, the studied isolates were initially given 24 h to produce a pre-formed biofilm (Bardbari et al., 2018). Following, the wells were gently discarded and washed three times with the normal saline solution. Then, 100 μL melittin (from 40 to 0.039 μg/μL), penicillin, and oxacillin (from 1,024 to 2 μg/μL) were added into the wells at the volume of 100 μL and incubated at 37°C for 24 h.

### Fractional biofilm inhibitory concentration index

*Staphylococcus aureus* isolates were cultured for 24 h at 37°C with shaking at 70 rpm in 5 mL of 1% Glu TSB. As mentioned above, the number of bacteria was adjusted to 0.5 McFarland standard. A suspension of 2 × 10^6^ CFUs as above prepared was added to each well of 96 Well microplate and incubated at overnight incubation, then the contents of the wells were slowly discarded, and the wells were washed three times with normal saline solution. the dilutions of each of melittin (from 40- to 0.039 μg/μL), penicillin (from 1,024 to 2 μg/μL), and oxacillin (from 1,024 to 2 μg/μL) were provided at a volume of 100 μL and added into the wells, and plates were incubated at 37°C for 24 h with shaking at 70 rpm, and as previously stated the quantity of biofilm was then measured. Then, MBIC was considered as discussed above, and FBICi for the two combined anti-bacterial agents was calculated as follows: FBICi = (MBIC drug A in combination/MBIC drug A alone) + (MBIC drug B in combination/MBIC drug B alone). FBIC indices are pointed to the kind of drug interaction if the following data are established: Synergy, values *n* ≤ 0.5; Partial synergy, values 0.5 < *n* < 1; Additive effect, for a value *n* = 1; Indifferent effect, for values 1 < *n* < 4; Antagonistic effect, for a value 4 ≤ n (Mackay et al., 2000).

### Fractional biofilm eradication concentration index

*Staphylococcus aureus* isolates were cultured for 24 h at 37°C with shaking at 70 rpm in 5 mL of 1% Glu TSB. As mentioned above, the number of bacteria was adjusted to 0.5 McFarland standard. A suspension of 2 × 10^6^ CFUs as above prepared was added to each well of 96 Well microplate and incubated at overnight incubation, then the contents of the wells were slowly discarded, and the wells were washed three times with normal saline solution. the dilutions of each of melittin (from 40 to 0.039 μg/μL), penicillin (from 1,024 to 2 μg/μL), and oxacillin (from 1,024 to 2 μg/μL) were provided at a volume of 100 μL and added into the wells, and plates were incubated at 37°C for 24 h with shaking at 70 rpm, and as previously stated, following three times of saline solution washing after discarding the contents of the wells, 100 μL of the solution was poured to the wells. After scratching and mixing, 10 μL of the well contents were cultured on MHA at 37°C for 24 h, and the growing colonies were counted. The MBEC values for melittin and penicillin, and oxacillin were defined as mentioned above. Afterward, FBECi for the two combined antibacterial agents was calculated as follows: FBECi = (MBEC drug A in combination/MBEC drug A alone) + (MBEC drug B in combination/MBEC drug B alone) (Mirzaei et al., 2022b). FBEC indices are pointed to the kind of drug interaction if the following data are established: Synergy, values *n* ≤ 0.5; Partial synergy, values 0.5 < *n* < 1; Additive effect, for a value *n* = 1; Indifferent effect, for values 1 < *n* < 4; Antagonistic effect, for a value 4 ≤ n (Mirzaei et al., 2022b).

### FE-SEM

Field emission scanning electron microscopy (FE-SEM) was used to visualize the effect of synergistic effects of melittin with penicillin and oxacillin antibiotics in their FBIC amounts on the biofilm formation of *S. aureus*. In this assay, we used one VRSA isolate. The sample preparation was performed based on the protocol previously described by Shams Khozani et al. (2019) with some modification. Briefly, the fresh bacteria were cultured in TSB with 1% glucose at 37°C for 24 h and then melittin, melittin-penicillin, and melittin-oxacillin was incubated with a bacterial suspension containing 1.5 × 10^7^ CFU/mL at 37°C for 24 h, in accordance with its predefined FBIC dose. Before incubation, sterile slides were cut and put into the wells. Briefly, the slides were gently washed three times with sterile distilled water and the sample was fixed in glutaraldehyde (2.5% in PBS 1 × ) for 3 h at room temperature. The slides were then rinsed three times in distilled water and post-fixed in 1.5% osmium tetroxide for 1 h. Then the slides were rinsed three times in distilled water, and they were dehydrated in a series of ethanol solutions (20–100%). The specimens were then mounted on conductive copper SEM tape for sample preparation, coated with gold nanoparticles, and then examined in an FE-SEM instrument (MIRA3, TESCAN Co., Czechia).

### Statistical analysis

Statistical analysis was conducted using SPSS Statistics (IBM SPSS Statistics for Windows, V.21.) to assess the data obtained from the experiments. To evaluate the significance of the findings, a paired-sample *t*-test was utilized, specifically comparing the concentrations of melittin and antibiotics in combination in terms of their anti-biofilm effect. Additionally, an ANOVA test was employed to compare values such as FIC, FBC, FBIC, and FBEC. The results were reported as the mean ± standard deviation (SD), unless specified otherwise. We conducted various tests to ensure the appropriateness of our data for statistical analysis. First, we assessed the normality of the data distribution using both the Shapiro-Wilk and Kolmogorov-Smirnov tests, and the data met the criteria for a Gaussian distribution. Next, for conducting *t*-tests, we verified several essential assumptions, including the scale of measurement, random sampling, normality of data distribution, adequacy of sample size, and equality of variance in standard deviation. All these prerequisites were confirmed before performing the *t*-tests. To use the ANOVA test, we ensured that each group’s sample was drawn from a population with a normal distribution, that all populations had a common variance, and that all samples were drawn independently of each other. Furthermore, we conducted Tukey’s *post-hoc* test to compare the mean of each group with the mean of every other group. The experiments were conducted with a confidence level of 95%, and a *p*-value less than 0.05 was considered statistically significant. To examine the correlation between examined concentrations and activity percentages, a non-linear regression test was performed. It is important to note that all experiments were repeated three times to ensure accuracy and reliability of the results.

## Results

### Antibacterial susceptibility pattern of VRSA, MRSA, and MSSA isolates

This study analyzed six samples from a total of 50 verified *S. aureus* isolates previously collected from patients with burn infections in a separate investigation (Bevalian et al., 2021). Additionally, two ATCC strains were included in the analysis. The results of the disk diffusion method used to test the antibacterial susceptibility of *S. aureus* isolates to gentamicin, clindamycin, rifampin, and DXT, as well as the MIC test for penicillin and oxacillin, are presented in Table 1.

### Antimicrobial findings of melittin alone and in combination with antibiotics toward planktonic form

This section presents the antimicrobial findings regarding the efficacy of melittin, both alone and in combination with penicillin, and oxacillin against the planktonic form of *S. aureus* to evaluate the potential synergistic effects of melittin and antibiotics in combating planktonic bacteria. To reach this aim, various experiments were conducted, including tests for MIC, MBC, FIC, and FBC. The effects of different concentrations of melittin, as well as melittin-antibiotic combinations, were examined.

### Determination of MIC, MBC, and MBC/MIC ratio

Six clinical isolates that collected from burn wound infections were tested for their MIC and MBC against penicillin, oxacillin, and melittin. Based on the MIC results, all of isolates resistant to penicillin (≥0.25 μg/mL) and 66% (*n* = 4) of isolates were resistant to oxacillin In addition, four of isolates were susceptible to vancomycin while unfortunately, we found two vancomycin-resistant MDR-MRSA isolate with MIC equal to 16 μg/ml. The findings indicated that melittin inhibited the growth of all *S. aureus* isolates, with MIC values ranging from 0.625 to 5 μg/ml. Besides, the results also showed the bactericidal activity for melittin against all *S. aureus* isolates, with MBC values ranging from 2.5 to 20 μg/ml.

For *S. aureus* isolates, the geometric means of the MIC values for penicillin, oxacillin, and melittin were 19.02, 16, and 1.62 μg/ml, respectively, and the geometric means of the MBC values for penicillin, oxacillin, and melittin were 107.63, 49.35, and 5.45 μg/ml, respectively. Also, the geometric means MBC/MIC ratios for penicillin, oxacillin, and melittin were 5.65, 3.08, 3.36 μg/ml respectively. Further details are displayed in Table 2.

**TABLE 2:** MIC, MBC, and MBC/MIC ratios of penicillin, oxacillin, and melittin against *S. aureus* strains.

Strains	Pen-MIC (μ g/mL)	Pen- MBC (μ g/mL)	Pen- MBC/ MIC ratio	Oxa- MIC (μ g/mL)	Oxa- MBC (μ g/mL)	Oxa- MBC/ MIC ratio	Mel- MIC (μ g/mL)	Mel- MBC (μ g/mL)	Mel- MBC/ MIC ratio
MSSA1	64	128	2	4	4	1	5	10	2
MRSA2	128	256	2	128	512	4	2.5	5	2
MSSA3	64	128	2	4	8	2	5	10	2
VRSA4	64	128	2	256	512	2	0.625	2.5	4
VRSA5	32	512	16	64	128	2	1.25	5	4
MRSA6	64	128	2	256	512	2	0.625	2.5	4
ATCC 25923	0.125	16	128	1	8	8	2.5	20	8
ATCC29213	2	32	16	0.5	8	16	0.625	2.5	4

MIC, minimum inhibitory concentration; MBC, minimum bactericidal concentration; ATCC, American type culture collection; pen: penicillin; oxa: oxacillin; mel: melittin.

### Synergistic results of planktonic bacteria

In the current investigation, we used the serial dilution method to determine the FICi and FBCi values, which stand for the interaction coefficients, to evaluate the antimicrobial agent interactions (Tables 3–6). These values indicate whether the combined inhibitory and bactericidal effects of drugs are synergistic, additive, indifferent, and/or antagonistic against selected isolates. In general, melittin-penicillin and melittin-oxacillin combination indexes based on FICi values against all isolates were 0.37 and 0.03, respectively. For MSSA 1, MRSA 2, MRSA 3, MRSA 4, MRSA 5, MRSA 6, ATCC 25923, and ATCC 29213, the geometric means of FICi for various melittin-penicillin synergistic concentrations were calculated as 1.25, 0.625, 1.25, 0.312, 0.312, 0.078, 0.625, and 0.078, respectively (Table 3). For MSSA 1, MRSA 2, MRSA 3, MRSA 4, MRSA 5, MRSA 6, ATCC 25923, and ATCC 29213, the geometric means of FICi for various melittin-oxacillin synergistic concentrations were calculated as 0.625, 0.038, 0.312, 0.002, 0.008, 0.004, 0.038, and 0.018, respectively (Table 4). A paired sample *t*-test revealed a significant difference between MIC values of melittin alone, melittin-penicillin, and melittin-oxacillin combinations against isolates (*p* = 0.006 and *p* = 0.005, respectively). In specifically, in MSSA 1, MRSA 2, MRSA 3, MRSA 4, MRSA 5, MRSA 6, ATCC 25923, and ATCC 29213 their MIC values of penicillin and melittin was decreased 8, 8, 8, 4, 8, 16, 8, and 16-fold, respectively, and for oxacillin and melittin was decreased 16, 128, 32, 256, 256, 256, 128, and 64-fold, respectively.

**TABLE 3:** The lowest synergistic concentrations of Penicillin -Melittin based on MIC against *S. aureus* strains.

MSSA1			MRSA2			MSSA3			VRSA4		
Pen + Mel (μ g/ml)	FIC indices	Drug interaction	Pen + Mel (μ g/ml)	FIC indices	Drug interaction	Pen + Mel (μ g/ml)	FIC indices	Drug interaction	Pen + Mel (μ g/ml)	FIC indices	Drug interaction
32 + 2.5	1	Additive	64 + 1.25	1	Additive	32 + 2.5	1	Additive	64 + 0.625	2	Indifferent
16 + 1.25	0.5	Synergism	32 + 0.625	0.5	Synergism	16 + 1.25	0.5	Synergism	32 + 0.312	0.999	Partial synergy
8 + 0.625	0.25	Synergism	16 + 0.312	0.25	Synergism	8 + 0.625	0.25	Synergism	16 + 0.156	0.499	Synergism
**VRSA5**			**MRSA6**			**ATCC25923**			**ATCC 29213**		
**Pen + Mel (μ g/ml)**	**FIC indices**	**Drug interaction**	**Pen + Mel (μ g/ml)**	**FIC indices**	**Drug interaction**	**Pen + Mel (μ g/ml)**	**FIC indices**	**Drug interaction**	**Pen + Mel (μ g/ml)**	**FIC indices**	**Drug interaction**
16 + 0.625	1	Additive	16 + 0.156	0.499	Synergism	0.0625 + 1.25	1	Additive	0.5 + 0.156	0.499	Synergism
8 + 0.312	0.499	Synergism	8 + 0.078	0.249	Synergism	0.0312 + 0.625	0.499	Synergism	0.25 + 0.078	0.249	Synergism
4 + 0.156	0.249	Synergism	4 + 0.039	0.124	Synergism	0.0156 + 0.312	0.249	Synergism	0.125 + 0.039	0.124	Synergism

FIC, fractional inhibitory concentration; ATCC, American type culture collection; pen: Penicillin; mel: Melittin.

**TABLE 4:** The lowest synergistic concentrations of Oxacillin -Melittin based on MIC against *S. aureus* strains.

MSSA1			MRSA 2			MSSA 3			VRSA 4		
Oxa + Mel (μ g/ml)	FIC indices	Drug interaction	Oxa + Mel (μ g/ml)	FIC indices	Drug interaction	Oxa + Mel (μ g/ml)	FIC indices	Drug interaction	Oxa + Mel (μ g/ml)	FIC indices	Drug interaction
1 + 1.25	0.5	Synergism	4 + 0.078	0.06245	Synergism	0.5 + 0.625	0.25	Synergism	2 + 0.004	0.014	Synergism
0.5 + 0.625	0.25	Synergism	2 + 0.039	0.031225	Synergism	0.25 + 0.312	0.1249	Synergism	1 + 0.002	0.007	Synergism
0.25 + 0.312	0.1249	Synergism	1 + 0.019	0.015413	Synergism	0.125 + 0.156	0.0624	Synergism	-	-	-
**VRSA 5**			**MRSA 6**			**ATCC 25923**			**ATCC 29213**		
**Oxa + Mel (μ g/ml)**	**FIC indices**	**Drug interaction**	**Oxa + Mel (μ g/ml)**	**FIC indices**	**Drug interaction**	**Oxa + Mel (μ g/ml)**	**FIC indices**	**Drug interaction**	**Oxa + Mel (μ g/ml)**	**FIC indices**	**Drug interaction**
1 + 0.019	0.030	Synergism	4 + 0.0097	0.030	Synergism	0.0312 + 0.078	0.0624	Synergism	0.0312 + 0.039	0.1249	Synergism
0.5 + 0.009	0.015	Synergism	2 + 0.004	0.0142	Synergism	0.0156 + 0.039	0.0312	Synergism	0.0156 + 0.019	0.0624	Synergism
0.25 + 0.004	0.0071	Synergism	1 + 0.002	0.0071	Synergism	0.0078 + 0.019	0.0156	Synergism	0.0078 + 0.009	0.030	Synergism

FIC, fractional inhibitory concentration; ATCC, American type culture collection; oxa: oxacillin; mel: melittin.

**TABLE 5:** The lowest synergistic concentrations of Penicillin -Melittin based on MBC against *S. aureus* strains.

MSSA1			MRSA2			MSSA3			VRSA4		
Pen + Mel (μ g/ml)	FBC indices	Drug interaction	Pen + Mel (μ g/ml)	FBC indices	Drug interaction	Pen + Mel (μ g/ml)	FBC indices	Drug interaction	Pen + Mel (μ g/ml)	FBC indices	Drug interaction
64 + 5	1	Additive	128 + 2.5	1	Additive	64 + 5	1	Additive	64 + 1.25	1	Additive
32 + 2.5	0.5	Synergism	64 + 1.25	0.5	Synergism	32 + 2.5	0.5	Synergism	32 + 0.625	0.5	Synergism
16 + 1.25	0.25	Synergism	32 + 0.625	0.25	Synergism	16 + 1.25	0.25	Synergism	16 + 0.312	0.25	Synergism
**VRSA5**			**MRSA6**			**ATCC 25923**			**ATCC 29213**		
**Pen + Mel (μ g/ml)**	**FBC indices**	**Drug interaction**	**Pen + Mel (μ g/ml)**	**FBC indices**	**Drug interaction**	**Pen + Mel (μ g/ml)**	**FBC indices**	**Drug interaction**	**Pen + Mel (μ g/ml)**	**FBC indices**	**Drug interaction**
128 + 1.25	0.5	Synergism	64 + 1.25	1	Additive	4 + 5	0.5	Synergism	16 + 1.25	1	Additive
64 + 0.625	0.25	Synergism	32 + 0.625	0.5	Synergism	2 + 2.5	0.25	Synergism	8 + 0.625	0.5	Synergism
32 + 0.312	0.125	Synergism	16 + 0.312	0.25	Synergism	1 + 1.25	0.125	Synergism	4 + 0.312	0.25	Synergism

FBC: fractional bactericidal concentration; ATCC: American type culture collection; pen: Penicillin; mel: Melittin.

**TABLE 6:** The lowest synergistic concentrations of Oxacillin -Melittin based on MBC against *S. aureus* strains.

MSSA 1			MRSA 2			MSSA 3			VRSA 4		
Oxa + Mel (μ g/ml)	FBC indices	Drug interaction	Oxa + Mel (μ g/ml)	FBC indices	Drug interaction	Oxa + Mel (μ g/ml)	FBC indices	Drug interaction	Oxa + Mel (μ g/ml)	FBC indices	Drug interaction
1 + 2.5	0.5	Synergism	256 + 2.5	1	Additive	1 + 1.25	0.25	Synergism	256 + 1.25	1	Additive
0.5 + 1.25	0.25	Synergism	128 + 1.25	0.5	Synergism	0.5 + 0.625	0.1249	Synergism	128 + 0.625	0.5	Synergism
0.25 + 0.625	0.1249	Synergism	64 + 0.625	0.25	Synergism	0.25 + 0.312	0.06245	Synergism	64 + 0.312	0.25	Synergism
**VRSA 5**			**MRSA 6**			**ATCC 25923**			**ATCC 29213**		
**Oxa + Mel (μ g/ml)**	**FBC indices**	**Drug interaction**	**Oxa + Mel (μ g/ml)**	**FBC indices**	**Drug interaction**	**Oxa + Mel (μ g/ml)**	**FBC indices**	**Drug interaction**	**Oxa + Mel (μ g/ml)**	**FBC indices**	**Drug interaction**
32 + 1.25	0.5	Synergism	256 + 1.25	1	Additive	1 + 2.5	0.25	Synergism	2 + 0.625	0.5	Synergism
16 + 0.625	0.25	Synergism	128 + 0.625	0.5	Synergism	0.5 + 1.25	0.1249	Synergism	1 + 0.312	0.25	Synergism
8 + 0.312	0.1249	Synergism	64 + 0.312	0.25	Synergism	0.25 + 0.625	0.06245	Synergism	0.5 + 0.156	0.1249	Synergism

FBC, fractional bactericidal concentration; ATCC, American type culture collection; oxa: oxacillin; mel: Melittin.

Most significantly, the interactions between melittin-penicillin and melittin-oxacillin combinations were assessed against all of isolates using MBC values. Melittin-penicillin and melittin-oxacillin generally had combination indexes based on FBC values against all isolates of 1.145 and 0.711, respectively. In this regard, for MSSA 1, MRSA 2, MSSA 3, MRSA 4, MRSA 5, MRSA 6, ATCC 25923, and ATCC 29213 strains the geometric means of FBCi for various melittin– penicillin synergistic concentrations were calculated as 2.5, 1.25, 2.5, 0.625, 0.625, 0.625, 2.5, and 0.625, respectively (Table 5), And, for MSSA 1, MRSA 2, MSSA 3, MRSA 4, MRSA 5, MRSA 6, ATCC 25923, and ATCC 29213 strains the geometric means of FBCi for various melittin– oxacillin synergistic concentrations were calculated as 1.25, 1.25, 0.625, 0.44, 0.625, 0.625, 1.25, and 0.312, respectively (Table 6). A paired sample *t*-test revealed a significant difference between MBC values of melittin alone, melittin-penicillin, and melittin-oxacillin combinations against isolates (*p* = 0.01 and *p* = 0.009, respectively). In specifically, in MSSA 1, MRSA 2, MRSA 3, MRSA 4, MRSA 5, MRSA 6, ATCC 25923, and ATCC 29213 their MBC values of penicillin and melittin was decreased 8, 8, 8, 8, 16, 8, 16, and 8-fold, respectively, and for oxacillin and melittin was decreased 16, 8, 32, 16, 16, 8, 32, and 16-fold, respectively.

### Biofilm formation assay and the kinetics of biofilm formation

**FIGURE 1:**
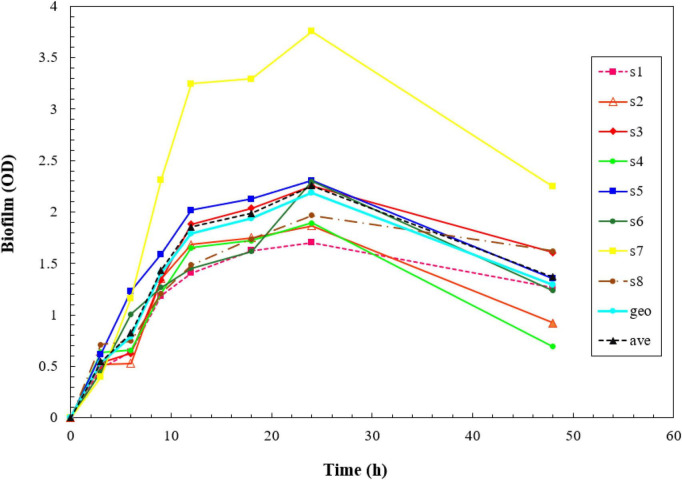
Kinetics of biofilm formation in the isolates over a 2- day-period using the crystal violet staining method. The results of the kinetics of biofilm formation showed that a maximum optical density (OD) value was reached after 48 h.

All the isolates showed strong biofilm formation, also the ATCC strain. The results of the kinetics of biofilm formation showed that a maximum OD value was reached after 24 h of incubation, which ranged between 1.703 and 3.757, and after 48 h, we saw a decrease in biofilm production, which ranged between 0.919 and 2.245. The geometric means of the OD value of biofilm formation for 3, 6, 9, 12, 18, 24, and 48 h were 0.533, 0.788, 1.398, 1.787, 1.934, 2.190, and 1.293, respectively. The results of the Kinetics of Biofilm Formation are shown in Table 7 and Figure 1.

**TABLE 7:** Kinetics of biofilm production capabilities of the *S. aureus* isolates.

Strain	Average OD 3 h	Standard deviation	Average OD 6 h	Standard deviation	Average OD 9 h	Standard deviation	Average OD 12 h	Standard deviation	Average OD 18 h	Standard deviation	Average OD 24 h	Standard deviation	Average OD 48 h	Standard deviation	Biofilm for-mation (24 h)
MSSA 1	0.47	0.087	0.64	0.194	1.18	0.26	1.407	0.107	1.62	0.049	1.703	0.232	1.26	0.254	Strong
MRSA 2	0.52	0.02	0.53	0.045	1.35	0.25	1.68	0.165	1.75	0.015	1.86	0.203	0.91	0.122	Strong
MSSA 3	0.53	0.02	0.62	0.089	1.33	0.186	1.88	0.041	2.03	0.188	2.25	0.601	1.6	0.211	Strong
VRSA 4	0.63	0.22	0.65	0.125	1.206	0.22	1.65	0.068	1.72	0.085	1.89	0.341	0.69	0.103	Strong
VRSA 5	0.61	0.1	1.23	0.142	1.59	0.048	2.01	0.149	2.12	0.041	2.3	0.263	1.35	0.242	Strong
MRSA 6	0.44	0.03	1.006	0.368	1.26	0.266	1.45	0.217	1.61	0.128	2.29	0.192	1.23	0.062	Strong
ATCC 25923	0.39	0.044	1.16	0.093	2.31	0.204	3.24	0.217	3.29	0.24	3.75	0.149	2.24	0.217	Strong
ATCC 29213	0.71	0.05	0.74	0.085	1.207	0.192	1.48	0.183	1.73	0.135	1.96	0.124	1.62	0.158	Strong
The geometric means	0.53		0.78		1.39		1.78		1.93		2.19		1.29		–
Average	0.54		0.82		1.43		1.85		1.98		2.25		1.36		–
Control negative	0.05		0.05		0.07		0.04		0.05		0.04		0.08		–

OD, optical density; ATCC, American type culture collection.

### Antibiofilm effects of melittin alone and in combination with antibiotics toward *S. aureus* biofilm

This section of our work presents the findings regarding the anti-biofilm effects of melittin, both individually and in combination with antibiotics, against *S. aureus* biofilms. The objective was to evaluate the potential synergistic effects of melittin and antibiotics in targeting biofilms, which are known for their heightened resistance to antimicrobial treatments. To accomplish this goal, a series of experiments were conducted, focusing on assessing the inhibition or eradication of biofilms. This involved evaluating parameters such as the MBIC and MBEC for melittin, penicillin, and oxacillin alone, as well as the combination of melittin with antibiotics, represented by the FBIC and FBEC. These experiments aimed to provide insights into the potential synergistic interactions between melittin and antibiotics, which could enhance their antibiofilm activities.

### Minimum biofilm inhibitory concentrations

The results showed that melittin inhibited the biofilm formation of all *S. aureus* isolates, with MBIC values ranging from 10 to 1.25 μg/mL. Besides, the MBIC results for penicillin and oxacillin were 512–516 μg/mL, and 512–532 μg/mL, respectively. Melittin, penicillin, and oxacillin had the geometric means of 3.85, 181.019, and 165.995 g/mL, respectively. Further details are depicted in Table 8.

**TABLE 8:** MBIC, MBEC, and MBEC/MBIC ratios of penicillin, oxacillin, and melittin against *S. aureus* strains.

Strains	Pen-MBIC (μ g/mL)	Pen- MBEC (μ g/mL)	Pen- MBEC/MBIC ratio	Oxa-MBIC (μ g/mL)	Oxa-MBEC (μ g/mL)	Oxa- MBEC/MBIC ratio	Mel- MBIC (μ g/mL)	Mel-MBEC (μ g/mL)	Mel- MBEC/MBIC ratio
MSSA1	128	1,024	8	128	512	4	5	20	4
MRSA2	512	512	1	256	1,024	4	10	10	1
MSSA3	512	1,024	2	128	256	2	5	40	8
VRSA4	512	1,024	2	512	1,024	2	1.25	20	16
VRSA5	128	>1,024	>8	256	512	2	5	20	4
MRSA6	512	1,024	2	512	1,024	2	2.5	10	4
ATCC 25923	16	256	16	64	256	4	5	20	4
ATCC 29213	64	512	8	32	128	4	2.5	20	8

MBIC, minimum biofilm inhibition concentration; MBEC, minimum biofilm eradication concentration; ATCC, American type culture collection; pen: penicillin; oxa: oxacillin; mel: melittin.

### Minimum biofilm eradication concentrations

The results showed that melittin eradicated all *S. aureus* isolates with MBEC values ranging from 40 to 10 μg/mL. Besides, the MBEC results for penicillin, and oxacillin were > 1,024–256 μg/mL, and > 1,024–128 μg/mL, respectively. The geometric means of the MBEC values for melittin, penicillin, and oxacillin, were 18.34, 689.1, and 469.506 μg/mL, respectively. The geometric means MBEC/MBIC ratios for melittin, penicillin, and oxacillin were 4.756, 3.622, 2.828 μg/ml. Further details are depicted in Table 8.

### Measurement of the synergistic effects

In the current study, we used the serial dilution method to determine whether the combined antibiofilm effects of antibacterial agents are synergistic, additive, indifferent, or antagonistic against all of isolates. To do this, we calculated the FBICi and FBECi, which are the interaction coefficients. In general, melittin-penicillin and melittin-oxacillin combination indexes based on FBIC values against all isolates were 0.23 and 0.177, respectively. Melittin-penicillin and melittin-oxacillin generally had combination indexes based on FBEC values against all isolates of 5 and 2.97, respectively.

The geometric means of the FBICi, and FBECi at various melittin– penicillin synergistic concentrations for strong biofilm-forming MRSA 1, 2, 3, 4, 5, 6, ATCC 25923, and ATCC 29213 were calculated as “0.625, 0.39, 0.386, 0.156, 0.883, 0.441, 0.441, and 0.312” for FBICi, and “3.535, 1.767, 14.142, 3.535, 7.071, 3.535, 7.071, and 7.071” for FBECi, respectively. The geometric means of the FBICi, and FBECi at various melittin– oxacillin synergistic concentrations for strong biofilm-forming MRSA 1, 2, 3, 4, 5, 6, ATCC 25923, and ATCC 29213 were calculated as “0.883, 1.767, 0.22, 0.11, 0.027, 0.027, 0.22, and 0.156” for FBICi, and “2.5, 2.5, 5, 2.5, 5, 2.5,2.5, and 2.5” for FBECi, respectively (Tables 9–12). A paired sample *t*-test revealed a significant difference between MBIC values of melittin alone, melittin-penicillin, and melittin-oxacillin combinations against isolates (*p* = 0.002 and *p* = 0.001, respectively), and in MBEC values of melittin alone, melittin-penicillin, and melittin-oxacillin combinations against isolates (*p* = 0.000 and *p* = 0.000, respectively).

**TABLE 9:** The lowest synergistic concentrations of Penicillin -Melittin based on MBIC against *S. aureus* strains.

MSSA1			MRSA 2			MSSA 3			VRSA 4		
Pen + Mel (μ g/ml)	FBIC indices	Drug interaction	Pen + Mel (μ g/ml)	FBIC indices	Drug interaction	Pen + Mel (μ g/ml)	FBIC indices	Drug interaction	Pen + Mel (μ g/ml)	FBIC indices	Drug interaction
32 + 1.25	0.5	Synergism	2 + 0.039	0.0078	Synergism	8 + 0.078	0.031225	Synergism	128 + 0.312	0.499	Synergism
16 + 0.625	0.25	Synergism	–	–	–	4 + 0.039	0.0156	Synergism	64 + 0.156	0.249	Synergism
8 + 0.312	0.1249	Synergism	–	–	–	2 + 0.019	0.0078	Synergism	32 + 0.078	0.1249	Synergism
**VRSA 5**			**MRSA 6**			**ATCC 25923**			**ATCC 29213**		
**Pen + Mel (μ g/ml)**	**FBIC indices**	**Drug interaction**	**Pen + Mel (μ g/ml)**	**FBIC indices**	**Drug interaction**	**Pen + Mel (μ g/ml)**	**FBIC indices**	**Drug interaction**	**Pen + Mel (μ g/ml)**	**FBIC indices**	**Drug interaction**
32 + 1.25	0.5	Synergism	128 + 0.625	0.5	Synergism	2 + 0.625	0.25	Synergism	16 + 0.625	0.5	Synergism
16 + 0.625	0.25	Synergism	64 + 0.312	0.249	Synergism	1 + 0.312	0.124	Synergism	8 + 0.312	0.25	Synergism
–	–	–	–	–	–	–	–	–	4 + 0.156	0.1249	Synergism

FBIC, fractional biofilm inhibitory concentration; ATCC, American type culture collection; pen: Penicillin; mel: Melittin.

**TABLE 10:** The lowest synergistic concentrations of Penicillin -Melittin based on MBEC against *S. aureus* strains.

MSSA 1			MRSA 2			MSSA 3			VRSA 4		
Pen + Mel (μ g/ml)	FBEC indices	Drug interaction	Pen + Mel (μ g/ml)	FBEC indices	Drug interaction	Pen + Mel (μ g/ml)	FBEC indices	Drug interaction	Pen + Mel (μ g/ml)	FBEC indices	Drug interaction
256 + 5	0.5	Synergism	128 + 2.5	0.5	Synergism	512 + 20	1	Additive	256 + 5	0.5	Synergism
128 + 2.5	0.25	Synergism	64 + 1.25	0.25	Synergism	256 + 10	0.5	Synergism	128 + 2.5	0.25	Synergism
**VRSA 5**			**MRSA 6**			**ATCC 25923**			**ATCC 29213**		
**Pen + Mel (μ g/ml)**	**FBEC indices**	**Drug interaction**	**Pen + Mel (μ g/ml)**	**FBEC indices**	**Drug interaction**	**Pen + Mel (μ g/ml)**	**FBEC indices**	**Drug interaction**	**Pen + Mel (μ g/ml)**	**FBEC indices**	**Drug interaction**
512 + 10	1	Additive	512 + 5	1	Additive	128 + 10	1	Additive	256 + 10	1	Additive
256 + 5	0.5	Synergism	256 + 2.5	0.5	Synergism	64 + 5	0.5	Synergism	128 + 5	0.5	Synergism

FBEC, fractional biofilm eradication concentration; ATCC, American type culture collection; pen: Penicillin; mel: Melittin.

**TABLE 11:** The lowest synergistic concentrations of Oxacillin -Melittin based on MBIC against *S. aureus* strains.

MSSA 1			MRSA 2			MSSA 3			VRSA 4		
Oxa + Mel (μ g/ml)	FBIC indices	Drug interaction	Oxa + Mel (μ g/ml)	FBIC indices	Drug interaction	Oxa + Mel (μ g/ml)	FBIC indices	Drug interaction	Oxa + Mel (μ g/ml)	FBIC indices	Drug interaction
32 + 1.25	0.5	Synergism	64 + 2.5	0.5	Synergism	8 + 0.312	0.1249	Synergism	64 + 0.156	0.249	Synergism
16 + 0.625	0.25	Synergism	32 + 1.25	0.25	Synergism	4 + 0.156	0.06245	Synergism	32 + 0.078	0.1249	Synergism
**VRSA 5**			**MRSA 6**			**ATCC 25923**			**ATCC 29213**		
**Oxa + Mel (μ g/ml)**	**FBIC indices**	**Drug interaction**	**Oxa + Mel (μ g/ml)**	**FBIC indices**	**Drug interaction**	**Oxa + Mel (μ g/ml)**	**FBIC indices**	**Drug interaction**	**Oxa + Mel (μ g/ml)**	**FBIC indices**	**Drug interaction**
1 + 0.019	0.0078	Synergism	4 + 0.019	0.0156	Synergism	2 + 0.156	0.06245	Synergism	2 + 0.156	0.1249	Synergism
–	–	–	–	–	–	–	–	–	1 + 0.078	0.06245	Synergism

FBIC, fractional biofilm inhibitory concentration; ATCC, American type culture collection; oxa: oxacillin; mel: Melittin.

**TABLE 12:** The lowest synergistic concentrations of Oxacillin -Melittin based on MBEC against *S. aureus* strains.

MSSA 1			MRSA 2			MSSA 3			VRSA 4		
Oxa + Mel (μ g/ml)	FBEC indices	Drug interaction	Oxa + Mel (μ g/ml)	FBEC indices	Drug interaction	Oxa + Mel (μ g/ml)	FBEC indices	Drug interaction	Oxa + Mel (μ g/ml)	FBEC indices	Drug interaction
128 + 5	0.5	Synergism	512 + 5	1	Additive	64 + 10	0.5	Synergism	256 + 5	0.5	Synergism
64 + 2.5	0.25	Synergism	256 + 2.5	0.5	Synergism	32 + 5	0.25	Synergism	128 + 2.5	0.25	Synergism
32 + 1.25	0.1249	Synergism	128 + 1.25	0.25	Synergism	16 + 2.5	0.1249	Synergism	64 + 1.25	0.1249	Synergism
VRSA 5			MRSA 6			ATCC 25923			ATCC 29213		
Pen + Mel (μ g/ml)	FBEC indices	Drug interaction	Pen + Mel (μ g/ml)	FBEC indices	Drug interaction	Pen + Mel (μ g/ml)	FBEC indices	Drug interaction	Pen + Mel (μ g/ml)	FBEC indices	Drug interaction
256 + 10	1	Additive	512 + 5	1	Additive	64 + 5	0.5	Synergism	32 + 5	0.5	Synergism
128 + 5	0.5	Synergism	256 + 2.5	0.5	Synergism	32 + 2.5	0.25	Synergism	16 + 02.5	0.25	Synergism
64.2.5	0.25	Synergism	128 + 1.25	0.25	Synergism	16 + 1.25	0.1249	Synergism	8 + 1.25	0.1249	Synergism

FBEC, fractional biofilm eradication concentration; ATCC, American type culture collection; oxa: oxacillin; mel: Melittin.

In specifically, in MSSA 1, MRSA 2, MRSA 3, MRSA 4, MRSA 5, MRSA 6, ATCC 25923, and ATCC 29213 their MBIC values of penicillin and melittin was decreased 16, 256, 256, 16, 8, 8, 16, and 16 -fold, respectively, and for oxacillin and melittin was decreased 8, 8, 32, 16, 256, 128, 32, and 32-fold, respectively. In MSSA 1, MRSA 2, MRSA 3, MRSA 4, MRSA 5, MRSA 6, ATCC 25923 and ATCC 29213 their MBEC values of penicillin and melittin was decreased 8, 8, 4, 8, 4, 4, 4, and 4 -fold, respectively, and their MBEC values of oxacillin and melittin was decreased 16, 8, 16, 16, 8, 8, 16, and 16-fold, respectively.

### FE-SEM

**FIGURE 2:**
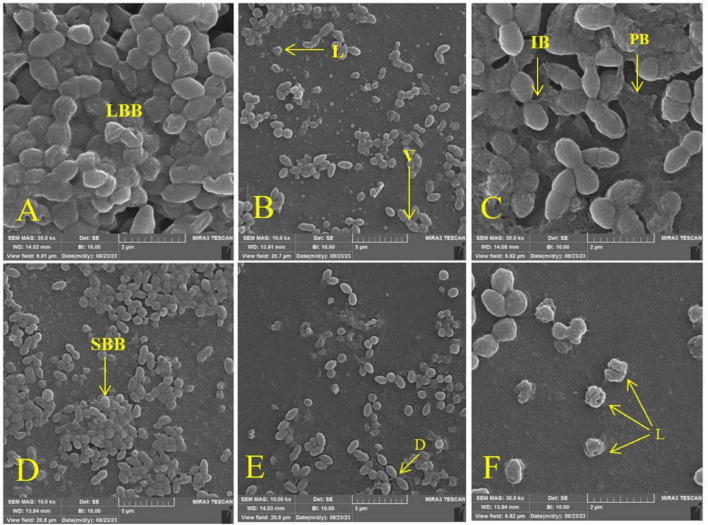
Effect of melittin, penicillin, and oxacillin, and their combination on biofilm biomass. **(A)** Biofilm formation (Untreated VRSA), **(B)** VRSA biofilm treated with 1.25 μg/ml melittin, **(C)** VRSA biofilm treated with 512 μg/ml penicillin, **(D)** VRSA biofilm treated with 512 μg/ml oxacillin, **(E)** VRSA biofilm treated with 0.312 μg/ml melittin and 128 μg/ml penicillin, **(F)** VRSA biofilm treated with 0.156 μg/ml melittin and 64 μg/ml oxacillin. LBB, large biofilm biomass; V, vesicle; L, lysis; IBB, interbacterial biofilm; PB, peripheral biofilm; SBB, small biofilm biomass; D, detachment.

The synergistic potential of melittin in eradicating or neutralizing biofilms, particularly against MDR-VRSA isolates, was assessed through the use of FE-SEM. The experimental outcomes unveiled that when melittin was combined with antibiotics at the concentration of FBICs, a remarkable and potent degradative effect was observed in biofilm structures. According to the results, melittin and antibiotics at the concentration of FBICs induced high destruction effects on the biofilm layer and interbacterial biofilm (IBB) and lysis of bacteria. Penicillin at the concentration of MBICs has less effect on biofilm destruction than melittin and oxacillin. Synergism of melittin-oxacillin at 0.156 μg/ml and 64 μg/ml induced more biofilm lysis compared to 0.312 μg/ml melittin and 128 μg/ml penicillin. Analysis of results showed that melittin not only has a significant biofilm degradation activity for removal of biofilm layers but is also able to kill them via invading their membranes (Figure 2).

## Discussion

The emergence of drug-resistant pathogens has posed a significant challenge in the field of infectious disease management, particularly with regards to the dwindling effectiveness of traditional antibiotics (Chinemerem Nwobodo et al., 2022). In recent years, AMPs have gained attention as potential alternatives due to their diverse mechanisms of action and ability to target drug-resistant bacteria (Mba and Nweze, 2022). Beta-lactam antibiotics, such as penicillin and oxacillin, have traditionally been used to inhibit bacterial cell wall synthesis (Bush and Bradford, 2016). However, the development of resistance mechanisms, such as the production of beta-lactamase enzymes, has rendered these antibiotics less effective against drug-resistant pathogens like MRSA (Munita and Arias, 2016). AMPs, known for their diverse mechanisms of action and broad-spectrum activity against bacteria, have emerged as potential candidates for combating drug-resistant pathogens (Mwangi et al., 2019). By combining AMPs with beta-lactam antibiotics, which work by inhibiting bacterial cell wall synthesis, it is possible to enhance the effectiveness of these conventional antibiotics against resistant strains.

To address the issue of MDR bacteria that produce biofilms, a comprehensive and flexible approach is necessary, using synergism between melittin and traditional antibiotics. Melittin is a promising AMP that effectively targets biofilms and their embedded bacteria, with multiple anti-biofilm mechanisms (Mirzaei et al., 2023). Our study aimed to investigate the effectiveness of melittin, alone and with antibiotics, against biofilm-producing MRSA and VRSA bacteria, focusing on preventing formation, promoting degradation, and killing embedded bacteria while reducing drug doses for safe application.

Our findings revealed that all *S. aureus* isolates exhibited robust biofilm formation, which is known to contribute to increased antibiotic resistance. To understand the kinetics of biofilm formation, we conducted experiments to monitor the growth and development of biofilms over time and our findings indicated that the bacteria reached substantial maximum biofilm biomass after 24 h. Based on these results, we selected this specific time point for our experimental investigation of biofilm-associated *S. aureus*.

Our results found that melittin was effective in inhibiting the growth of all tested isolates of *S. aureus*, with MIC values ranging from 0.625 to 5 μg/ml. Additionally, the results showed that melittin had bactericidal activity against all *S. aureus* isolates, with MBC values ranging from 2.5 to 10 μg/ml. These findings are consistent with previous studies that have also reported the antimicrobial activity of melittin against *S. aureus*. Mirzaei et al. (2023) found that melittin was effective in inhibiting the growth of MDR-methicillin resistant *Staphylococcus aureus* (MRSA), with MIC values ranging from 0.625–2.5 μg/ml. Another study by Hakimi Alni et al. (2020) reported that melittin had potent bactericidal activity against planktonic *S. aureus*, with MBC values ranging from 4 to 16 μg/ml.

These results have also been obtained in other studies conducted by Khozani et al. (2019) and Lima et al. (2021). In a study by Lima et al. (2021) melittin killed *S. aureus* cells at concentrations ranging from 0.12 to 16 μM.

Most importantly, we evaluated the interaction between antimicrobial agents based on their MIC and MBC value, and hence, FICi and FBCi were determined. The analysis of this test will be crucial for the combination of antibacterial medicines due to the fact that the MIC and MBC concentrations are the concentrations needed to inhibit or eradicate drug-resistant bacteria. Besides, the value of the geometric mean of FICi and FBCi of the melittin-penicillin synergistic concentrations were 0.37 and 1.14, respectively and the value of the geometric mean of FICi and FBCi for melittin-oxacillin synergistic concentrations were 0.03 and 0.71, respectively. These findings show that FICi of the melittin-penicillin in these isolates can have a synergistic effect, while FBCi of the melittin-penicillin in these isolates can have an indifferent effect and FICi of the melittin- oxacillin in these isolates can have a synergistic effect, while FBCi of the melittin- oxacillin in these isolates can have a partial synergy effect.

By means of the determination of FICi and FBCi for antimicrobial agents, the precise information about their bacteriostatic effect can be deduced in combination. There are numerous instances where AMPs can drastically lower the antibiotic effective concentration. The peptides primarily function at the bacterial cell wall, facilitating the drug’s access to its intracellular target. The exopolysaccharide layer of biofilm communities can be penetrated by AMPs in addition, or they may even stop bacterial adherence and biofilm formation. Other peptides that can interfere with community quorum-sensing processes or directly prevent the establishment of bacterial resistance mechanisms. Of note, lower peptide concentrations are required for adjuvant function and bacterial resistance suppression than are necessary for direct antibacterial action. Recent research in animal models demonstrates that AMPs can be administered safely for innovative combination chemotherapies and function as adjuvants at non-toxic dosages (Li et al., 2020). In this study, due to the fact that both penicillin and oxacillin drugs affect the bacterial cell wall and, on the other hand, the mechanism of the main effect of melittin peptide is also on the cell wall, the results show that they strengthen each other’s effect.

Infections caused by MRSA are an issue of long-standing global concern (Goering et al., 2019). The results of this study show that the simultaneous use of oxacillin and melittin can have promising results, thus preventing the increase of resistance to this drug, and as a result, it is not necessary to look for stronger and more harmful drugs for treatment. In a studies conducted by Wangai et al. (2019), the results shows that from a total of 187 *S. aureus* isolates revealed an overall MRSA prevalence of 53.4%. Also in the study that do by Hussein et al. (2019), 50.4% of isolates were MRSA. Considering the importance of MRSA in different countries, application of synergism can be promising.

Biofilms, which are collections of bacteria adhered to abiotic and biotic surfaces and encased in a self-produced extracellular matrix, pose an additional challenge (Schulze et al., 2021). The formation of biofilms and entrapment of cells in a polymer-based matrix reduces the sensitivity of bacteria to antimicrobial compounds, making it difficult to eradicate infections (Mohamad et al., 2023). To address this issue, discovering new classes of antibiotics with diverse mechanisms of action has become a top priority. Studies have demonstrated that AMPs show promise as potential candidates for the development of novel antibiofilm medications. These peptides exhibit diverse mechanisms to combat biofilms, including the inhibition, obstruction, and eradication of preexisting biofilms (Luong et al., 2020). Particularly, melittin, an AMP derived from bee venom, has exhibited significant antibacterial and antibiofilm properties (Ko et al., 2020). Accordingly, another aim of the present study was to investigate the impact of melittin, alone and in combination with penicillin and oxacillin antibiotics, on biofilm-forming MDR-MRSA bacteria. The study aimed to assess its ability to inhibition of biofilm formation, promote the degradation of existing biofilms, and eliminate the bacteria residing within the biofilms. The results of MBIC and MBEC showed the excellent activity of melittin against the biofilms of all *S. aureus* isolates. The mode MBIC values of melittin against all isolates was 5 μg/mL and the MBEC values was from 20 μg/mL. In comparison to melittin, the results of MBIC and MBEC for penicillin and oxacillin showed a weak effect against the biofilm of *S. aureus* isolates. In this regard, MBIC results for penicillin and oxacillin were 512 μg/mL, and 128 μg/mL, respectively. Additionally, MBEC results for penicillin, and oxacillin were 1,024 μg/mL, and 1,024 μg/mL, respectively. The findings of our study provide further evidence supporting the classification of melittin as an anti-biofilm peptide (ABP). It has been found that AMPs possess multiple overlapping mechanisms that combat biofilms, such as targeting the bacterial membrane within biofilms, breaking down the polysaccharide and biofilm structure, and suppressing the genes responsible for biofilm formation (Yasir et al., 2018).

Furthermore, when compared to planktonic bacteria, traditional antibiotics show significantly a reduced efficacy against biofilm-forming bacteria. This is primarily due to the presence of a protective matrix composed of polysaccharides, which confers greater resistance to antibiotics (Singh et al., 2021). Consequently, combination therapy involving multiple antibiotics is commonly employed to mitigate the development of resistance to individual antimicrobial agents and take advantage of their synergistic effects (Tängdén, 2014). In the context of bacterial biofilms, combination treatment is particularly attractive since the heterogeneous nature of biofilm formation necessitates targeting cells in various metabolic stages, including those in exponential growth and latent states (Khan et al., 2021). By combining multiple agents that target different components of the biofilm, it becomes possible to effectively eliminate the biofilm structure. Our results also found a synergistic effect of melittin in combination with antibiotics toward biofilm-forming MDR-MRSA and VRSA. The geometric mean values for melittin–penicillin and melittin– oxacillin concentrations based on FBICi against *S. aureus* were 0.23 and 0.177, respectively. Some reports noted melittin for its synergistic effect on biofilm when used with antibiotics. Hakimi Alni et al. (2020) found that synergistic growth-inhibitory effects of mupirocin with melittin could be considered as a promising approach in the treatment of MRSA and VRSA isolates. Besides, the geometric mean values of FBECi for melittin–penicillin and melittin– oxacillin concentrations against *S. aureus* were 5 and 2.973, respectively. Our findings indicate that the FBIC values of the melittin-penicillin and melittin-oxacillin combinations in these isolates exhibit a synergistic effect. In contrast, the FBEC values of the melittin-penicillin and melittin-oxacillin combinations display antagonistic and indifferent effects, respectively. There are several reports in the literature regarding the synergistic action of melittin with commercial antibiotics against MDR bacterial strains. In a study described by Mirzaei et al. (2023) on strong biofilm of MDR-MRSA and -*Pseudomonas aeruginosa* as well as studies by others on biofilm-forming MDR *P. aeruginosa* (Khozani et al., 2019) the synergistic effects of melittin with antibiotics were found that are similar to our report. Also, Pereira et al. (2020) showed that the combination of melittin with oxacillin has a synergistic activity on MRSA strains.

Finally, based on previous research about melittin and findings from antibiotics, the mechanism for the synergism between melittin with penicillin and oxacillin can be included in the following area. (1) Cell membrane disruption: melittin is known for its ability to disrupt bacterial cell membranes. It forms pores or channels within the lipid bilayer, leading to increased permeability (Shai, 1999). This initial action can enhance the penetration of antibiotics into bacterial cells. (2) Enhanced antibiotic entry: penicillin and oxacillin are beta-lactam antibiotics that inhibit cell wall synthesis in bacteria. By creating breaches in the cell membrane, melittin may facilitate greater access of these antibiotics to their target site within the bacterial cell (Stojowska-Swędrzyńska et al., 2023). (3) Synergistic antibacterial activity: when used in combination with melittin, penicillin, and oxacillin, the disruption of the cell membrane by melittin can weaken the structural integrity of bacteria, making them more susceptible to the cell wall-targeting antibiotics. (4) Reduced antibiotic resistance: bacterial resistance often arises due to limited antibiotic entry or altered drug targets. Melittin’s action of increasing membrane permeability can counteract these resistance mechanisms, potentially restoring the effectiveness of penicillin and oxacillin against antibiotic-resistant strains. In summary, the synergy between melittin and antibiotics like penicillin and oxacillin can be attributed to melittin’s cell membrane-disrupting properties, which enhance antibiotic entry, counter antibiotic resistance mechanisms, and result in additive or synergistic antibacterial effects. This combination strategy holds promise for combating bacterial infections more effectively, especially against resistant strains. The dual action of melittin and antibiotics was found to induce extensive disruption within the biofilm matrix. This combination exhibited an exceptional ability to permeate the bacterial membrane of VRSA strains, resulting in a cascade of transformative events. The FE-SEM micrographs showed a myriad of structural alterations within the bacterial biofilm, including: (1) Wrinkle formation: The biofilm structure exhibited pronounced wrinkling, indicative of a profound impact on its integrity. (2) Cell splitting: melittin and antibiotics induced the splitting of bacterial cells, leading to a significant reduction in the biofilm’s structural integrity. (3) Vesiculation: the formation of vesicles indicated cellular stress and structural damage. (4) Lysis: pronounced lysis or cell rupture was evident, further confirming the effectiveness of the combination therapy. (5) Perforation: the bacterial membrane exhibited perforations, which indicated the disruption of cellular boundaries. (6) Disruption: the biofilm experienced significant disruption, resulting in the loss of its cohesive structure. (7) Membrane detachment: the bacterial membrane detachment, particularly in response to the melittin-antibiotic combination, highlighted the profound impact on cellular membranes. These observations, as visually represented in the Figure 2, underscore the potential of melittin in synergy with antibiotics to wreak havoc on MDR-VRSA biofilms. The combination therapy’s multi-pronged assault on the biofilm structure, ranging from membrane penetration to structural deformities, holds significant promise as a strategy to combat the tenacity of these resilient biofilms. Further research into the precise mechanisms and optimization of this approach may pave the way for innovative solutions to address biofilm-related infections, especially those involving antibiotic-resistant VRSA strains.

Our findings show that antimicrobial resistance, and the exopolysaccharide matrix do affect the therapeutically achievable doses of antimicrobial medicines in biofilm-associated bacteria. This excellent sort of bacterial resistance, known as biofilm-associated resistance, is a result of biofilms’ inactive lifestyle. Our study demonstrated the potential of combining melittin with beta-lactam antibiotics to combat MRSA and VRSA. Additionally, the combination of melittin and beta-lactam antibiotics has shown a synergistic effect, where the antimicrobial activity is significantly enhanced compared to the individual components alone.

## Conclusion

The rising challenge of drug-resistant pathogens, especially the diminishing efficacy of traditional antibiotics, has prompted the exploration of alternative strategies. In conclusion, our research investigated the anti-biofilm potential of melittin alone and in combination with penicillin and oxacillin against MDR-MRSA and -VRSA. The emergence of drug-resistant pathogens has posed significant challenges in infectious disease management, leading to the dwindling effectiveness of traditional antibiotics. AMPs, particularly melittin, have shown promise as potential alternatives due to their diverse mechanisms of action and ability to target drug-resistant bacteria. Our study demonstrated that melittin effectively inhibited the growth of all tested isolates of *S. aureus*, with MIC values ranging from 0.625 to 5 μg/ml, and exhibited bactericidal activity with MBC values ranging from 2.5 to 10 μg/ml. Furthermore, the combination of melittin with beta-lactam antibiotics, penicillin, and oxacillin, showed a synergistic effect, significantly enhancing the antimicrobial activity compared to individual components alone. This synergistic approach offers a potential solution to combat biofilm-forming MDR-MRSA and -VRSA, preventing the increase of resistance to conventional antibiotics and reducing the need for stronger and more harmful drugs.

While our study highlights the promising potential of melittin and combination therapy, there are several avenues for future research in this field. Firstly, further investigations into the mechanisms of action of melittin and its interactions with beta-lactam antibiotics within biofilms are warranted. Understanding these processes at a molecular level can provide crucial insights into developing more effective treatment strategies. Moreover, *in vivo* studies are essential to evaluate the safety and efficacy of melittin and combination therapy in animal models before proceeding to human clinical trials. Assessing the potential toxicities, pharmacokinetics, and optimal dosing regimens will be crucial for translating these findings into practical therapeutic interventions. Additionally, exploring the effects of melittin and combination therapy on biofilms formed by other drug-resistant bacterial species will broaden the applicability of this approach. Different bacteria may exhibit varying responses to the treatment, and understanding these variations can lead to tailored therapeutic strategies for specific infections. Lastly, investigating the long-term effects of melittin and combination therapy on biofilm prevention and eradication will be essential to assess the potential for resistance development over time. This information will guide the development of sustainable treatment approaches that can withstand the evolution of bacterial resistance. In conclusion, our research offers a promising direction in the fight against drug-resistant infections by harnessing the potential of melittin and combination therapy. With further research and development, we hope that this approach will contribute to the return of effectiveness to once-ineffective antibiotics and pave the way for novel and sustainable treatment options for multidrug-resistant pathogens. In summary, our research provides compelling evidence supporting the efficacy of melittin against planktonic and strong MRSA and VRSA biofilms. Furthermore, we observed significant synergistic effects when melittin was combined with penicillin and oxacillin. These findings suggest that melittin, in combination with conventional antibiotics, holds great promise as a potential therapeutic candidate for *in vivo* infections caused by MDR bacteria. Additionally, melittin has shown the potential to restore the effectiveness of penicillin and oxacillin antibiotics in treating MDR infections. The application of AMPs, such as melittin, to revitalize beta-lactam antibiotics against MRSA and VRSA, represents an innovative and promising approach in combating antibiotic-resistant bacteria. Further research is warranted to optimize dosages, understand the mechanisms of melittin, and explore its interactions with beta-lactam antibiotics to ensure successful clinical applications.

## Data availability statement

The raw data supporting the conclusions of this article will be made available by the authors, without undue reservation.

## Author contributions

SJ: Data curation, Formal analysis, Investigation, Methodology, Software, Writing—original draft, Writing—review and editing. SR: Methodology, Writing—review and editing. RM: Software, Writing—review and editing. GI: Supervision, Writing—review and editing. KP: Methodology, Supervision, Validation, Writing—review and editing.
