# Supplementary material for: A hope for ineffective antibiotics to return to treatment: investigating the anti-biofilm potential of melittin alone and in combination with penicillin and oxacillin against multidrug resistant-MRSA and -VRSA
Source: Front Microbiol. 2024 Feb 1;14:1269392. doi: 10.3389/fmicb.2023.1269392 (PMC10870424; doi:10.3389/fmicb.2023.1269392)
Supplement: Supplementary file 1 [file Table_1.DOCX]

Table 1. The primers used in this study for detection of *S. aureus*.

| Target gene | Sequence primer (5^/^ ➔3^/^) | Ref |
| --- | --- | --- |
| 16srRNA | F: TCGTGTCGTGAGATGTTGGGTTA  R: GGTTTCGCTGCCCTTTGTATTGT | (1) |
| nuc A | F: CTG GCA TAT GTA TGG CAA TTG TT  R: TAT TGA CCT GAA TCA GCG TTG TCT | (2) |

1. Zhang S, Wang P, Shi X, Tan H. Inhibitory properties of Chinese Herbal Formula SanHuang decoction on biofilm formation by antibiotic-resistant Staphylococcal strains. Sci Rep. 2021;11(1):7134.

2. Abbasi-Montazeri E, Khosravi AD, Feizabadi MM, Goodarzi H, Khoramrooz SS, Mirzaii M, et al. The prevalence of methicillin resistant Staphylococcus aureus (MRSA) isolates with high-level mupirocin resistance from patients and personnel in a burn center. Burns. 2013;39(4):650-4.
